# Madicolous Chironomidae from the Brazilian Atlantic Forest: a checklist with notes on altitudinal distributions (Diptera, Insecta)

**DOI:** 10.3897/zookeys.751.20611

**Published:** 2018-04-19

**Authors:** Erika Mayumi Shimabukuro, Susana Trivinho-Strixino

**Affiliations:** 1 Centro de Ciências da Natureza – CCN, Universidade Federal de São Carlos, Campus Lagoa do Sino. Rod. Lauri Simões de Barros, Km 12, Bairro Aracaçu, Buri, São Paulo, Brazil. CEP.: 18290-000; 2 Laboratório de Ecologia de Insetos Aquáticos, Depto. de Hidrobiologia, Universidade Federal de São Carlos, Rodovia Washington Luiz, São Carlos, São Paulo, Brazil, CEP.:13565-905

**Keywords:** hygropetric habitats, mountains, non-biting midges, semi-aquatic habitats, tropical forest

## Abstract

Thin layers of water running over rocky surfaces are characteristic of madicolous habitats, which harbor a peculiar Chironomidae community. However, information on the identity, distribution, and ecology of madicolous chironomids in the Neotropical region are still sparse. The main purpose of this research is to reveal and contribute to the ecology of madicolous Chironomidae species, especially regarding their altitudinal distribution in the Atlantic Forest. Sampling was performed using our own designed emergence traps deployed from 0 to 2700 m a.s.l. in 70 sites in three mountains in southeastern Brazil. Sixty taxa of chironomids were collected and identified, of which only 22 are known to science. Most of the species showed a wider distribution than previously known, both in terms of geographic and altitudinal ranges, while others showed significant association with particular altitudinal bands (as evidenced by the indicator species analysis). Atlantic Forest mountainous regions are known to harbor one of the richest fauna in the world and have been suffering from several types of environmental impacts, including climate change, which will especially affect taxa living in specialized habitats. The narrow range of tolerance to environmental conditions verified for mountain species, and the fact that many of them are rare and endemic, make the conservation efforts in these areas indispensable.

## Introduction

Madicolous habitats are characterized by a thin layer of water that frequently flows over rocky surfaces, and for this reason they are also known as hygropetric habitats. The first to use the term “hygropetrischen” was Thienemann in 1909, when studying the biology of trichopterans from Central Europe. Throughout the twentieth century, some catalogues of madicolous fauna were done in North America (Sinclair and Marshall 1987) and Europe (Bertrand 1948, Vaillant 1956). More recently, most of the progress done on the study of madicolous organisms came out of taxonomic works (Sinclair 1988, Cranston 1998, Roque and Trivinho-Strixino 2004, Short 2009, Short et al. 2013, Bilton 2015, Pinho and Andersen 2015, Trivinho-Strixino and Shimabukuro 2017, Shimabukuro et al. 2017a, b, Pinho and Shimabukuro 2018), emphasizing the potential of this habitat in harboring a rich and endemic overlooked fauna. In South America, madicolous habitats have recently provided remarkable discoveries on the occurrence of insects, from new records (Roque and Trivinho-Strixino 2004, Short et al. 2013, Pinho and Andersen 2015) to several new species (Pepinelli et al. 2009, Silva et al. 2012, Trivinho-Strixino et al. 2012, Miller and Montano 2014, Shimabukuro et al. 2017a, b, Pinho and Shimabukuro 2018).

In natural ecosystems, madicolous insects can live in a wide range of habitats, such as shoreline of streams or in isolated overflowing groundwater. Additionally, when robust water bodies like streams and lakes are scarce, for example on mountaintops, madicolous biotopes can be the only source of permanent water allowing aquatic and semi-aquatic insects to establish themselves and survive, contributing to the maintenance of biodiversity in natural systems. The true madicolous inhabitants (eumadicoles) present morphological and physiological adaptations favoring their survival in such a specific environmental condition, as seen by the presence of strong locomotor appendages to hang on the rocky substrate in larval stages (Trivinho-Strixino et al. 2012), presence of strong hooks on the pupal abdomen and the production of silk by the larvae (Boothroyd 2005) or living inside portable cases to avoid water carrying (Fittkau and Reiss 1998).

The Chironomidae family is one of the most diverse within Diptera. Species numbers reach an estimated 20,000 (Coffman 1995), though only 6,000 approximately have been described. This remarkable evolutionary success allowed them to occur in all zoogeographic regions, including Antarctica, tolerating even the harshest environmental conditions (Sugg et al. 1983, Linevich 1971, Watanabe et al. 2006, Andersen et al. 2016a). Although the immature stages of known species show high dependence on water (Ferrington 2008), some are semi-aquatic or terrestrial, and researchers have recorded some in artificial madicolous systems (Cranston 1984, Boothroyd 2005, Hamerlík et al. 2010).

A high diversity of chironomids is expected to occur in natural madicolous habitats from tropical regions, but this biotope has so far been neglected in freshwater researches, making it difficult to have an estimate on the diversity of insects living in such habitats. Furthermore, concerning the taxonomy of chironomids, most of the descriptions are based exclusively on adult males, making it difficult to obtain the information on the habitats, behavior, and other ecological information related to aquatic stages.

Despite significant progress on Chironomidae research in the last decade (Trivinho-Strixino 2011, Mendes et al. 2007a), most registered species are still concentrated in Nearctic and Palearctic regions, which emphasize the urgent need for studies in Tropical regions that present potentially higher diversity. In this research, the first checklist is provided of madicolous Chironomidae from the Atlantic Forest, which is one of the richest hotspots in the world, and still the most affected by habitat loss (Myers et al. 2000). In addition, notes on distribution in the altitudinal gradients and other ecological features are included.

## Methods

### Study area

The exceptional biodiversity verified in the Brazilian Atlantic Forest is mainly due to the environmental heterogeneity owed to its singular geographical characteristics. Specifically, the region is characterized by a high variation in latitudinal (originally from parallels 3° to 30°) and altitudinal ranges (0 to 2892 m a.s.l.). These generate a diverse forest composition and a wide variety of habitats (Ribeiro et al. 2009). Also, the high precipitation rates recorded annually (about 1400 mm) (Forti et al. 2005) that are allied to the abundant and easily found overflowing groundwater, provide a perfect condition for maintaining different kinds of water bodies. More specifically, madicolous habitats thrive even in the upmost sites.

**Figure 1.**
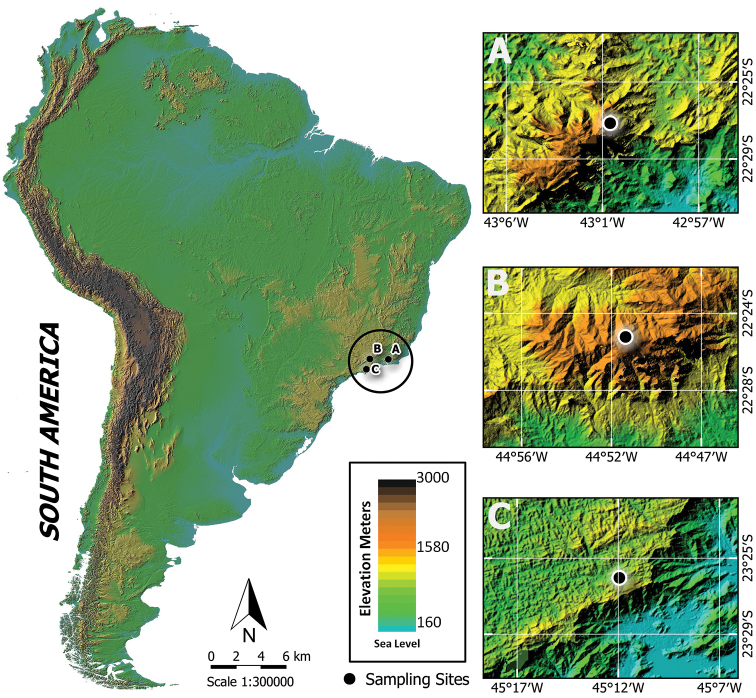
Localization of the study area comprehending the complete altitudinal range in the Atlantic Forest (Brazil). **A** Maximum altitudinal range in PARNASO **B** Maximum altitudinal range in APASM **C** Maximum altitudinal range in PESM.

This study was conducted inside three conservation units from the Atlantic Forest in southeastern Brazil: Serra do Mar State Park (PESM), Serra dos Órgãos National Park (PARNASO), and Serra da Mantiqueira Environmental Protection Area (APASM) (mean distance between those areas is 185 km) (Figure 1). These specific locations were chosen because they present the utmost variation in topographic profiles in the Atlantic Forest, which allowed us to properly explore madicolous Chironomidae communities in a wide range of altitudes (from 0 to 2700 m a.s.l.).

PESM is the widest continuous protected area inside the Atlantic Forest (332,000 ha). It encompasses the whole coast of São Paulo State, including stretches at the sea level and some peaks that elevate the altitudinal range up to 1270 m a.s.l. The vegetation present in the region includes: mangroves, “restinga” (costal dunes), costal vegetation, ombrophilous dense forest, and “campos de altitude” (high altitude-vegetation composed predominantly of grass, shrub, and herbaceous vegetation). In this locality, sites between 0–1100 m a.s.l. were explored (Figure 1C).

PARNASO is in the mountainous region of Rio de Janeiro State, where it occupies 20,024 ha. The region’s relief is marked with slopes, which generates a high altitudinal gradient. The upmost site in the park reaches 2263 m a.s.l., and the vegetation changes alongside variations in elevation: submontane forest, montane forest, misty forest, and campos de altitude. In this park, intermediate altitudes were explored, between 1200–2100 m a.s.l. (Figure 1A).

APASM includes three states from the Southeast: São Paulo, Rio de Janeiro, and Minas Gerais, comprising an area of 421,804 ha. It harbors two of the five highest mountains in Brazil, including the culminant site at 2798 m a.s.l. (Pedra da Mina Mountain). The vegetation in the region creates a mosaic of phytophysiognomies, composed by upper-montane forests, araucaria forests and campos de altitude. In this locality, sites from 1700 to 2700 m a.s.l. were sampled (Figure 1B).

### Sampling

Madicolous habitats were sampled every 200–300 m along the gradient. At least three replicates were obtained in each altitudinal band.

**Figure 2.**
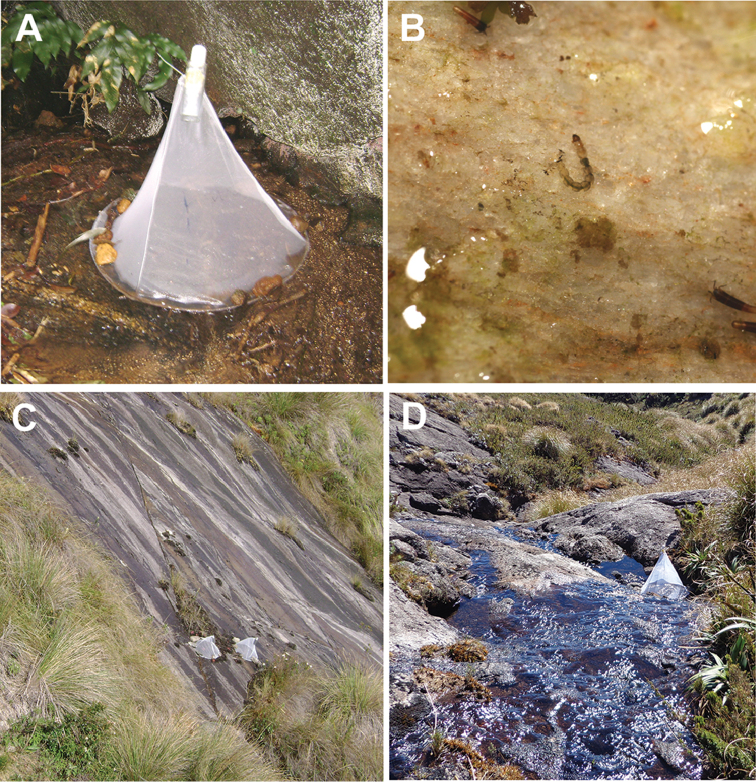
Field images. **A** The emergence trap installed above a madicolous system **B** Madicolous larva in natural habitat (*Podonomus* sp.) **C** Example of madicolous system characterized by seepage on rocky substrate **D** Example of madicolous system created at the stream bank.

The adult sampling was performed with emergence traps (Shimabukuro et al. 2015), that was left in the field for 7 days (Figure 2A). The advantage of using this type of trap is that it guarantees that the emergent adults really belong to the interested habitat, once the immature have completed their development at that specific site. In addition, the isolation provided by this trap prevents invasion by foreign specimens. From the substrate below the traps, larvae, pupae, and exuviae (Figure 2B) were also collected with a hand net. Organisms were preserved in absolute ethanol and slide mounted with Euparal before analyzing them in optic microscopy. Only male adults were identified to species level. When the specimens did not match any species'description, probably representing new species, they were designated as morphotypes.

### Data analysis

For each species found, a set of data from the literature was added, including previous information on their geographical distribution, altitudinal records, habitats and development stages and sexes so far known. After the literature data, the “Remarks” include the data obtained by us: 1. type of madicolous habitat where the species was found, such as stream edge (Figure 2C) or isolated rocky outflow (Figure 2D); 2. latitudinal and altitudinal records; 3. environmental data (water temperature, pH, dissolved oxygen, and canopy cover); 4. locality where the species was recorded (PESM, PARNASO, and APASM); and 5. altitudinal specificity depicted by the species. In addition, some information about the morphospecies (probably new species) found was included, hereafter denominated as “unknown species”.

Information on the development stages, habitat, and locality of each species and morphospecies recorded in our study is summarized in Table 1 (supplementary material). When the development stage consists only of adults (A), it does not mean that immature specimens were not collected; rather, that the association between adult and immature stages were not yet established. In addition, some taxa were recorded only from its immature instars (I).

In order to test the degree of each species'affinity within the altitude in which they occurred, an indicator species analysis (Dufrêne and Legendre 1997) was applied and the “indicator value” (IndVal) and “p-value” obtained for each species; the IndVal varies from 0 to 1.0, and higher values indicate a more expressive altitudinal representation. Significant indicator species presented p<0.05, although p = 0.06 species were considered as high altitudinal specificity, 0.06<p<0.2 species with median altitudinal specificity, and p>0.2 species with low altitudinal specificity. This analysis was performed in R Cran Project 3.0.3 (R Core Development) software using the “labdsv” package (Roberts 2007).

## Results

A total of 60 species, including 22 known species and 38 morphospecies, was recorded, as follows:

### SUBFAMILY CHIRONOMINAE

#### TRIBE CHIRONOMINI

##### *Claudiotendipes* Andersen, Mendes & Pinho, 2017

Two valid species; Neotropical; running water.

###### C. froehlichi

Taxon classificationAnimaliaDipteraChironomidae

Andersen, Mendes & Pinho, 2017

####### Distribution.

BRAZIL – Camacan, Bahia State (15°23'28"S, 39°33'56"W; 15°23'02"S, 39°34'10"W; 15°23'10"S, 39°34'03"W) Rio de Janeiro State (11°53'40"S, 45°36'06"W), Salesópolis, São Paulo State; Campos do Jordão, São Paulo State (22°41'40"S, 45°27'36"W); Grão-Pará, Santa Catarina State (28°11'28"S, 49°23'30"W); Urubici, Santa Catarina State (28°01'41"S, 49°22'36"W).

####### Elevation.

508–1014 m a.s.l.

####### Habitats.

Low-order streams.

####### Known stages.

L, P, M.

####### References.

Andersen et al. (2017).

####### Remarks.

*C. froehlichi* was found on marginal rocks of small streams, at 206 m a.s.l. (23°27'52"S, 45°11'55"W) and 1444 m a.s.l. (22°26'51"S, 43°00'48"W), extending the altitudinal range of the species. Environmental characterization: Water temperature 10–17 °C; dissolved oxygen 9.0–9.2 mg.l^-1^; pH 5.0–5.5; dense canopy cover. The species was found in PESM (São Paulo State) and PARNASO (Rio de Janeiro State). Low altitudinal specificity (IndVal: 0.17; p = 0.84).

##### *Lauterborniella* Thienemann & Bause, 1913

One valid species; Holartic, Nearctic, and Neotropical; standing water.

**Unknown species.** *Lauterborniella* sp. 1. Locality: PESM. Altitudinal record: 1080 m a.s.l. Significant altitudinal specificity (IndVal: 0.5; **p = 0.02**).

##### *Nilothauma* Kieffer, 1921

52 valid species; worldwide; running and stagnant water.

**Unknown species.** *Nilothauma* sp. 1 Locality: APASM. Altitudinal record: 1570 m a.s.l. Low altitudinal specificity (IndVal: 0.14; p = 1.00).

##### *Oukuriella* Epler, 1986

24 valid species; Neotropical, associated with freshwater sponges and submerged wood.

###### O. sublettei

Taxon classificationAnimaliaDipteraChironomidae

Messias & Oliveira, 1998

####### Distribution.

BRAZIL – Paru do Oeste River, missão Cururu, Amazonas State; Parque Estadual de Campos do Jordão, São Paulo State (22°41'40"S, 45°27'36"W).

####### Elevation.

20–1600 m a.s.l.

####### Habitats.

Larvae found in submerged wood in a rocky first-order stream.

####### Known stages.

L, P, M.

####### References.

Messias and Oliveira (1998); Fusari et al. (2013); Bellodi et al. (2016).

####### Remarks.

*O. sublettei* was found on marginal rocks of small streams, at 745 m a.s.l. Environmental characterization: Water temperature 17 °C; dissolved oxygen 8.2 mg.l^-1^; pH 5.5; fast flowing; dense vegetal canopy (more than 80% covered). The species was found in PESM (São Paulo State). Median altitudinal specificity (IndVal: 0.33; p = 0.14).

##### *Polypedilum* Kieffer, 1912

More than 440 valid species; worldwide; standing and running water.

###### P. solimoes

Taxon classificationAnimaliaDipteraChironomidae

Bidawid-Kafka, 1996

####### Distribution.

BRAZIL – Tarumã River, Amazonas State; Florianópolis, Santa Catarina State (27°28'05"S, 48°22'58"W), UCAD, Santa Catarina State (27°31'51"S, 48°30'44"W), Santinho Beach, Santa Catarina State (27°27'S, 48°23'W).

####### Elevation.

20–80 m a.s.l.

####### Habitats.

Adults collected close to a large Amazonian river. Larva found in leaf packs and detritus associated with the following bromeliad species: *Aechmea lindeni* (E. Morren) Baker, *Canistrum lindenii* (Regel) Mez, *Neoregelia laevis* (Mez) L.B. Smith, *Nidularium innocentii* Lem., *Vriesea philippocoburgii* Wawra, and *V. vagans* (L.B. Smith) L.B. Smith.

####### Known stages.

L, P, F, M.

####### References.

Bidawid-Kafka (1996); Pinho et al. (2013).

####### Remarks.

*P. solimoes* was found on marginal rocks of small stream, at 1570 m a.s.l., extending the elevation records of this species. Environmental characterization: Water temperature 16.3 °C; dissolved oxygen 8.4 mg.l^-1^; pH 6.4; slow flowing; vegetal canopy absent. The species was found in APASM (Minas Gerais State). Low altitudinal specificity (IndVal: 0.14; p = 1.0).

**Unknown species.** *Polypedilum* (s. str.) sp. 1. Locality: APASM. Altitudinal record: 1570 m a.s.l. Low altitudinal specificity (IndVal: 0.14; p = 1.00); *Polypedilum* (s. str.) sp. 2 Locality: PESM. Altitudinal record: 1080 m a.s.l. High altitudinal specificity (IndVal: 0.33; p = 0.06); *Polypedilum* (s. str.) sp. 3. Locality: PARNASO. Altitudinal record: 2125 m a.s.l. Low altitudinal specificity (IndVal: 0.17; p = 0.84); Polypedilum (Pentapedilum) sp. 1; Locality: PESM. Altitudinal record: 200 m a.s.l. Median altitudinal specificity (IndVal: 0.33; p = 0.15). Polypedilum (Pentapedilum) sp. 2. Locality: PARNASO. Altitudinal record: 2125 m a.s.l. Low altitudinal specificity (IndVal: 0.17; 6 p = 0.85). Polypedilum (Tripodura) sp. 1. Locality: PESM. Altitudinal record: 25 m a.s.l. Low altitudinal specificity (IndVal: 0.25; p = 0.33).

##### *Stenochironomus* Kieffer, 1919

101 valid species; worldwide; miners of living or dead vegetal tissue.

**Unknown species.** *Stenochironomus* sp. 1. Locality: PESM. Altitudinal record: 1075 m a.s.l. Low altitudinal specificity (IndVal: 0.17; p = 0.84); *Stenochironomus* sp. 2. Locality: PESM. Altitudinal record: 70 m a.s.l. Low altitudinal specificity (IndVal: 0.25; p = 0.32).

#### TRIBE PSEUDOCHIRONOMINI

##### *Pseudochironomus* Thienemann, 1934

13 valid species; Nearctic, Palearctic, Neotropical; running and standing water.

###### P. ruah

Taxon classificationAnimaliaDipteraChironomidae

Shimabukuro & Trivinho-Strixino, 2017

####### Remarks.

*P. ruah* was recorded for the first time on marginal rocks of a small stream, at 2575 m a.s.l. (22°25'41"S, 44°50'38"W), in APASM (Minas Gerais State). Larva, pupa, female and male have been described by Shimabukuro et al. (2017a). Environmental characterization: Water temperature 22 °C; dissolved oxygen 7.4 mg.l^-1^; pH 5.5; fast flowing; vegetal canopy absent. High altitudinal specificity (IndVal: 0.67; **p = 0.003**).

#### TRIBE TANYTARSINI

##### *Paratanytarsus* Thienemann & Bause, 1913

45 valid species; worldwide; running and stagnant water

###### P. silentii

Taxon classificationAnimaliaDipteraChironomidae

Trivinho-Strixino, 2010

####### Distribution.

BRAZIL – Parque Estadual do Jaraguá, São Paulo State (23°24'S, 45°44'W).

####### Elevation.

800 m a.s.l.

####### Habitats.

Stream.

####### Known stages.

M.

####### References.

Trivinho-Strixino (2010).

####### Remarks.

*P. silentii* was found on marginal rocks of small streams, from 200–1445 m a.s.l., extending the altitudinal range of the species. Environmental characterization: Water temperature varied from 10–21 °C; dissolved oxygen 7.9–9.9 mg.l^-1^; pH 5–5.5; slow to fast flowing; occurring in scarce vegetal canopy to dense coverage (30–70% covered). The species was found in PARNASO (Rio de Janeiro State) and PESM (São Paulo State). Low altitudinal specificity (IndVal: 0.24; p = 0.25).

#### *Tanytarsus* van der Wulp, 1874

More than 300 valid species; worldwide; aquatic and terrestrial

##### T. alaidae

Taxon classificationAnimaliaDipteraChironomidae

Trivinho-Strixino & Shimabukuro, 2017

###### Remarks.

*T. alaidae* was recorded for the first time on marginal rocks of a small stream, at 2575 m a.s.l. (22°25'41"S, 44°50'38"W), in APASM (Minas Gerais State). At present, only adult males have been described (Trivinho-Strixino and Shimabukuro 2017). Environmental characterization: Water temperature 22 °C; dissolved oxygen 7.4 mg.l^-1^; pH 5.5; fast flowing; vegetal canopy absent. Low altitudinal specificity (IndVal: 0.17; p = 0.82).

##### T. alienus

Taxon classificationAnimaliaDipteraChironomidae

Trivinho-Strixino & Shimabukuro, 2017

###### Remarks.

*T. alienus* was recorded for the first time on marginal rocks of a small stream, at 2575 m a.s.l. (22°25'41"S, 44°50'38"W), in APASM (Minas Gerais State). At present, only adult males have been described (Trivinho-Strixino and Shimabukuro 2017). Environmental characterization: Water temperature 22 °C; dissolved oxygen 7.4 mg.l^-1^; pH 5.5; fast flowing; vegetal canopy absent. Low altitudinal specificity (IndVal: 0.17; p = 0.85).

##### T. angelae

Taxon classificationAnimaliaDipteraChironomidae

Trivinho-Strixino & Shimabukuro, 2017

###### Remarks.

*T. angelae* male adults were recorded in a temporary pool at 2218 m a.s.l. (22°24'697"S, 44°50'93"W) in APASM (Minas Gerais State), in a rocky fountain at 2126 m a.s.l. (22°27'60.5'S, 43°01'68.9"W) in PARNASO (Rio de Janeiro State), and in the same locality at 1444 (rocky substrate marginal to a stream – 22°26'84.8'S, 43°00'79.8"W). At present, only adult males have been described (Trivinho-Strixino and Shimabukuro 2017). Environmental characterization: Water temperature 10–12 °C; dissolved oxygen 8.8–9.0 mg.l^-1^; pH 5.0; partial to absent canopy cover. Low altitudinal specificity (IndVal: 0.17; p = 0.85).

##### T. digitatus

Taxon classificationAnimaliaDipteraChironomidae

Sanseverino & Fittkau, 2006

###### Distribution.

BRAZIL – Paquequer River, Teresópolis, Rio de Janeiro State.

###### Elevation.

1100 m a.s.l.

###### Habitats.

Adults collected in light trap close to a first-order stream.

###### Known stages.

M.

###### References.

Sanseverino and Fittkau (2006).

###### Remarks.

*T. digitatus* was found on marginal rocks of small streams, at 25 m (23°28'20.72"S, 45°11'38.43"W) and 1445 m a.s.l (22°26'84.8"S, 43°00'79.8"W), extending the altitudinal range of the species. Environmental characterization: Water temperature 10–19.5 °C; dissolved oxygen 8.1–9.0 mg.l^-1^; pH 5.0; slow flowing; dense canopy cover. The species was found in PESM (São Paulo State) and PARNASO (Rio de Janeiro State). Low altitudinal specificity (IndVal: 0.5; **p = 0.02).**

##### T. giovannii

Taxon classificationAnimaliaDipteraChironomidae

Sanseverino & Trivinho-Strixino, 2010

###### Distribution.

BRAZIL – São Carlos, São Paulo State (21°59'10"S, 47°52'32"W; 21°58'16"S, 47°53'14"W; 21°59'21.4"S, 47°51'14.2"W); Corumbá, Mato grosso do Sul State (19°34'30.06"S, 57°00'52.4"W).

###### Elevation.

90–850 m a.s.l.

###### Habitats.

Low-order streams and eutrophic lake.

###### Known stages.

L, P, M.

###### References.

Sanseverino and Trivinho-Strixino (2010); Trivinho-Strixino et al. (2015).

###### Remarks.

*T. giovannii* was found on marginal rocks of small streams, at 2575 m a.s.l., extending the altitudinal range of the species. Environmental characterization: Water temperature 22 °C; dissolved oxygen 7.4 mg.l^-1^; pH 5.5; fast flowing; vegetal canopy absent. The species was found in APASM (Minas Gerais State). Low altitudinal specificity (IndVal: 0.17; p = 0.84).

### SUBFAMILY ORTHOCLADIINAE

#### *Corynoneura* Winertz, 1846

Approximately 96 valid species; worldwide; running and stagnant waters.

##### C. unicapsulata

Taxon classificationAnimaliaDipteraChironomidae

Wiedenbrug & Trivinho-Strixino, 2011

###### Distribution.

BRAZIL – Parque Estadual do Jaraguá, São Paulo State (23°27'S, 46° 45'W) Serra do Japi, Jundiaí, São Paulo State (23°14'30"S, 46°57'16"W); Paraisópolis, Minas Gerais State (22°39'54.81"S, 45°55'38.29"W); São Luís do Purunã, Paraná State (25°27.180'S, 49°43.435'W); BRAZIL, Serra da Bodoquena, Mato Grosso do Sul State (20°41'49"S, 56°52'54"W); Alto Paraíso de Goiás, Goiás State (14°9'34.92"S, 47°35'37.32W); COSTA RICA, Caccao, Guanacaste.

###### Elevation.

750–1370 m a.s.l.

###### Habitats.

Litter in stones marginal to small streams.

###### Known stages.

L, P, F, M.

###### References.

Wiedenbrug and Trivinho-Strixino (2011); Wiedenbrug et al. (2012).

###### Remarks.

*C. unicapsulata* was found on marginal rocks of small streams, at 2575 m a.s.l., extending its altitudinal occurrence. Environmental characterization: Water temperature 22 °C; dissolved oxygen 7.4 mg.l^-1^; pH 5.5; slow flow to stagnant; vegetal canopy absent (shrub-herbaceous vegetation. The species was found in APASM (Minas Gerais State). Low altitudinal specificity (IndVal: 0.17; p = 0.85).

##### C. hermanni

Taxon classificationAnimaliaDipteraChironomidae

Wiedenbrug & Trivinho-Strixino, 2011

###### Distribution.

BRAZIL – Ubatuba, São Paulo State (23°30.468'S, 45°11.923'W and 23°30.789'S, 45°14.442'W)

###### Elevation.

0–60 m a.s.l.

###### Habitats.

Litter standing near the water surface from small streams.

###### Known stages.

L, P, F, M.

###### References.

Wiedenbrug and Trivinho-Strixino (2011); Wiedenbrug et al. (2012).

###### Remarks.

*C. hermanni* was found on marginal rocks of small streams and rocky seepages (large exposed rock in the middle of the forest), at 1570–1580 m a.s.l., extending the altitudinal occurrence of this species. Environmental characterization: Water temperature 15–16.3 °C; dissolved oxygen 8.4–10.2 mg.l^-1^; pH 5.0–6.4; slow to fast flowing; vegetal canopy completely absent. The species was found in APASM (Minas Gerais State) and PARNASO (Rio de Janeiro State), extending slightly the geographical distribution of this species. Low altitudinal specificity (IndVal: 0.29; p = 0.23).

##### C. septadentata

Taxon classificationAnimaliaDipteraChironomidae

Wiedenbrug & Trivinho-Strixino, 2011

###### Distribution.

BRAZIL – Parque Estadual do Jaraguá, São Paulo State (23°27'S, 46°45'W), Nova Friburgo, Rio de janeiro State, Rio Boa Vista; Bocaina de Minas, Minas Gerais State (22°19'S, 44°34'W); Serra do Japi, Jundiaí, São Paulo State (23°14'30"S, 46°57'16"W); Serra da Bodoquena, Mato Grosso do Sul State (20°41'49"S, 56°52'54"W).

###### Elevation.

700–1200 m a.s.l.

###### Habitats.

Litter near the water surface of a small shallow stream.

###### Known stages.

L, P, F, M.

###### References.

Wiedenbrug and Trivinho-Strixino (2011); Wiedenbrug et al. (2012).

###### Remarks.

*C. septadentata* was found on marginal rocks of small streams and on rocky seepages (large exposed rock in the middle of the forest), from 1045–1580 m a.s.l., extending slightly the altitudinal range of this species. Environmental characterization: Water temperature 15–17 °C; dissolved oxygen 8.6–10.2 mg.l^-1^; pH 5; moderate to fast flowing; vegetal canopy reduced or absent (less than 30% covered). The species was found in PARNASO (Rio de Janeiro State) and PESM (São Paulo State). Low altitudinal specificity (IndVal: 0.09; p = 0.88).

##### C. sertaodaquina

Taxon classificationAnimaliaDipteraChironomidae

Wiedenbrug & Trivinho-Strixino, 2011

###### Distribution.

BRAZIL – PESN, Ubatuba, São Paulo State (23°30.789'S, 45°14.442'W; 23°31.068'S, 45°14.845'W; 23°31.231'S, 45°14.625'W); Alto Paraíso de Goiás, Goiás State (14°9'34.92"S, 47°35'37.32"W); Serra da Bodoquena, Mato Grosso do Sul State (20°41'49"S, 56°52'54"W), São Simão, São Paulo State; São Luís do Purunha, Paraná State (25°27.180'S, 49°43.435'W).

###### Elevation.

20–1020 m a.s.l.

###### Habitats.

Surface of stones of shallow fast flowing waters and also in litter near the water surface of streams.

###### Known stages.

L, P, F, M.

###### References.

Wiedenbrug and Trivinho-Strixino (2011); Wiedenbrug et al. (2012).

###### Remarks.

*C. sertaodaquina* was found on marginal rocks of small streams, at 70 m a.s.l. Environmental characterization: Water temperature 17 °C; dissolved oxygen 9.2 mg.l^-1^; pH 5.5; wet substrate, without any flow; reduced vegetal canopy (about 20% covered). The species was found in PESM (São Paulo State). Low altitudinal specificity (IndVal: 0.25; p = 0.32).

#### *Onconeura* Andersen & Seather, 2005

Seven valid species; Neotropical and Nearctic; running and stagnant water.

##### O. japi

Taxon classificationAnimaliaDipteraChironomidae

Wiedenbrug, Mendes, Pepinelli & Trivinho-Strixino, 2009

###### Distribution.

BRAZIL, Serra do Japi, Jundiaí, São Paulo State (23°14'38"S, 46°57'02"W); PESM, Ubatuba, São Paulo State (23°30.46'S, 45°11.923'W and 23°30.789'S, 45°14.442'W).

###### Elevation.

1058 m a.s.l.

###### Habitats.

Litter below a waterfall of a first-order stream.

###### Known stages.

L, P, F, M.

###### References.

Wiedenbrug et al. (2009).

###### Remarks.

*O. japi* was found on marginal rocks of small streams, at 1570 m a.s.l., extending the altitudinal record of the species. Environmental characterization: Water temperature 16.3 °C; dissolved oxygen 8.4 mg.l^-1^; pH 6.4; slow flowing; vegetal canopy completely absent. The species was found in APASM (Minas Gerais State), extending the geographical distribution of the species. Low altitudinal specificity (IndVal: 0.14; p = 1.0).

##### O. oncovolsella

Taxon classificationAnimaliaDipteraChironomidae

Wiedenbrug, Mendes, Pepinelli & Trivinho-Strixino, 2009

###### Distribution.

BRAZIL – PESM, Ubatuba, São Paulo State (23°31.068'S, 45°14.845'W; 23°31.087'S, 45°14.621'W; 23°30.789'S, 45°14.442'W); São Francisco de Paula, Rio Grande do Sul State.

###### Elevation.

23–61 m a.s.l.

###### Habitats.

Surface of stones in fast flowing waters.

###### Known stages.

L, P, F, M.

###### References.

Wiedenbrug et al. (2009).

###### Remarks.

*O. oncovolsella* was found on marginal rocks of small streams, at 1445 m a.s.l., extending the altitudinal record of the species. Environmental characterization: Water temperature 10 °C; dissolved oxygen 9 mg.l^-1^; pH 5; fast flowing; partial vegetal canopy (50% covered). The species was found in PARNASO (Rio de Janeiro State). Low altitudinal specificity (IndVal: 0.25; p = 0.31).

**Unknown species.** *Onconeura* sp. 1. Locality: PARNASO. Altitudinal record: 1445 m a.s.l. Low altitudinal specificity (IndVal: 0.25; p = 0.30); *Onconeura* sp. 2 Locality: PARNASO. Altitudinal record: 1445 m a.s.l. Low altitudinal specificity (IndVal: 0.25; p = 0.32); *Onconeura* sp. 3 Locality: PESM. Altitudinal record: 1085 m a.s.l. Low altitudinal specificity (IndVal: 0.17; p = 0.83).

#### *Thienemanniella* Kieffer, 1911

53 valid species; worldwide; running and stagnant water.

**Unknown species.** *Thienemanniella* sp.1. Locality: APASM. Altitudinal record: 1570 m a.s.l. Low altitudinal specificity (IndVal: 0.14; p = 1.00).

#### *Bryophaenocladius* Thienemann, 1934

115 valid species; worldwide; terrestrial and semi-terrestrial, few aquatic.

##### B. carus

Taxon classificationAnimaliaDipteraChironomidae

(Roback, 1962)

###### Distribution.

BRAZIL – Parque Estadual Intervales, Iporanga, São Paulo State (24°30'S, 48°37'W); PANAMA, Canal Zone, Curundu, Holbrook Air Force Base.

###### Elevation.

20–100 m a.s.l.

###### Habitats.

Mosses on tree barks.

###### Known stages.

L, P, F, M.

###### References.

Roback (1962); Sæther (1976); Spies and Reiss (1996); Sæther (1981); Wang et al. (2006); Donato (2011).

###### Remarks.

*B. carus* was found on marginal rocks of small streams, at 1075 m a.s.l., extending the altitudinal range of the species. Environmental characterization: Water temperature 15.8 °C; dissolved oxygen 8.1 mg.l^-1^; pH 5; moderate to fast flowing; dense vegetal canopy (more than 70% covered). The species was found in PESM (São Paulo State). Low altitudinal specificity (IndVal: 0.17; p = 0.85).

**Unknown species.** *Bryophaenocladius* sp. 1. Locality: PESM. Altitudinal record: 1075 m a.s.l. Low altitudinal specificity (IndVal: 0.17; p = 0.87).

#### *Caaporangonbera* Andersen, Pinho & Mendes, 2015

Four valid species; Brazil, Atlantic Forest; unknown habitat, but possibly terrestrial or semi-terrestrial.

##### C. intervales

Taxon classificationAnimaliaDipteraChironomidae

Andersen, Pinho & Mendes, 2015

###### Distribution.

BRAZIL – Parque Estadual Intervales, Ribeirão Grande, São Paulo State (24°15'S, 48°10'W).

###### Elevation.

500 m a.s.l.

###### Habitats.

Unknown, but possibly terrestrial or semi-terrestrial.

###### Known stages.

M.

###### References.

Andersen et al. (2015b).

###### Remarks.

*C. intervales* was found on marginal rocks of small streams, at 740 m a.s.l., extending slightly the altitudinal range of this species. Environmental characterization: Water temperature 15.7 °C; dissolved oxygen 9.4 mg.l^-1^; pH 5.5; very slow flowing; partial vegetal canopy (50% covered). The species was found in PESM (São Paulo State). Median altitudinal specificity (IndVal: 0.33; p = 0.14).

#### *Cricotopus* van der Wulp, 1874

218 valid species; worldwide; running and standing water.

**Unknown species.** *Cricotopus* sp. 1. Locality: APASM. Altitudinal record: 2575 m a.s.l.. Low altitudinal specificity (IndVal: 0.17; p = 0.85); *Cricotopus* sp. 2 Locality: APASM and PARNASO. Altitudinal range: 1445–1570 m a.s.l. Low altitudinal specificity (IndVal: 0.15; p = 0.47); *Cricotopus* sp. 3 Locality: PESM. Altitudinal range: 70–1075 m a.s.l. Low altitudinal specificity (IndVal: 0.13; p = 0.58); *Cricotopus* sp. 4 Locality: PESM. Altitudinal range: 70–200 m a.s.l.. Significant altitudinal specificity (**IndVal: 0.71; p = 0.003)**; *Cricotopus* sp. 5 Locality: APASM. Altitudinal range: 1750 m a.s.l. Low altitudinal specificity (IndVal: 0.14; p = 1.0).

#### *Limnophyes* Eaton, 1875

91 valid species; worldwide, except for Oceania and Antarctic; aquatic, terrestrial and semi-terrestrial habitats.

##### L. guarani

Taxon classificationAnimaliaDipteraChironomidae

Pinho & Andersen, 2015

###### Distribution.

BRAZIL – Serra do Corvo Branco, Grão-Pará, Santa Catarina State (28°03'21"S, 49°22'00"W).

###### Elevation.

1241 m a.s.l.

###### Habitats.

Madicolous habitat.

###### Known stages.

L, P, F, M.

###### References.

Pinho and Andersen (2015).

###### Remarks.

*L. guarani* was found on rocky seepages and also on marginal rocks of first order streams, from 1570–2700 m a.s.l., extending the altitudinal range of the species. Environmental characterization: Water temperature 16.3–22 °C; dissolved oxygen 6.4–8.4 mg.l^-1^; pH 5.5–6.4; slow to fast flowing; vegetal coverage completely absent (shrub-herbaceous vegetation). The species was found in APASM (Minas Gerais State), extending the geographical records to northernmost. Median altitudinal specificity (IndVal: 0.19; p = 0.18).

##### L. gercinoi

Taxon classificationAnimaliaDipteraChironomidae

(Oliveira, Messias & Santos, 1995)

###### Distribution.

BRAZIL – Parque João Paulo II, Curitiba, Paraná State; UCAD, Florianópolis, Santa Catarina State; Parque Nacional de São Joaquim, Urubici, Santa Catarina State (28°07'32"S, 49°29'38"W); Nova Teutônia, Santa Catarina State (27°11'S, 52°23'W).

###### Elevation.

300–1822 m a.s.l.

###### Habitats.

Adults collected with entomological net and malaise trap close to stream.

###### Known stages.

F, M.

###### References.

Oliveira et al. (1995); Spies and Reiss (1996); Mendes et al. (2007b); Roque et al. (2007).

###### Remarks.

*L. gercinoi* was found on marginal rocks of small streams, from 1080–1445 m a.s.l. Environmental characterization: Water temperature 10.0–16.1 °C; dissolved oxygen 8.1–9.9 mg.l^-1^; pH 5; slow to fast flowing; partial canopy (about 50–70% covered). The species was found in PARNASO (Rio de Janeiro State), PESM (São Paulo State) extending the geographical records to northernmost. Low altitudinal specificity (IndVal: 0.19; p = 0.2).

**Unknown species.** *Limnophyes* sp. 1. Locality: PARNASO. Altitudinal range: 1445–2125 m a.s.l. Low altitudinal specificity (IndVal: 0.11; p = 0.87).

#### *Lipurometriocnemus* Saether, 1981

Four valid species; Nearctic and Neotropical; unknown habitat, probably semi-aquatic and terrestrial.

##### L. biancae

Taxon classificationAnimaliaDipteraChironomidae

Andersen, Pinho & Mendes, 2016

###### Distribution.

BRAZIL – Parque Nacional de São Joaquim, Urubici, Santa Catarina State (28°07'37"S, 49°28'47"W).

###### Elevation.

1670 m a.s.l.

###### Habitats.

Male collected in malaise trap in cloud forest.

###### Known stages.

M.

###### References.

Andersen et al. (2016b).

###### Remarks.

*L. biancae* was found on marginal rocks of low order streams and small waterfalls, extending the altitudinal records from 1570–2575 m a.s.l. Environmental characterization: Water temperature varied from 11–22 °C; dissolved oxygen 7.4–10.0 mg.l^-1^; pH 4.5–6.4; slow to fast flowing; vegetal coverage completely absent (shrub-herbaceous vegetation). The species was found in PARNASO (Rio de Janeiro State) and APASM (Minas Gerais State), the northernmost records. Low altitudinal specificity (IndVal: 0.16; p = 0.39).

**Unknown species.** *Lipurometriocnemus* sp. Locality: APASM. Altitudinal record: 2700 m a.s.l. Low altitudinal specificity (IndVal: 0.2; p = 0.45).

#### *Metriocnemus* van der Wulp, 1874

67 valid species, worldwide, except for Oceania and Antarctic; mosses, Phytotelmata, springs, ditches, streams, lakes, and rock pools.

**Unknown species.** *Metriocnemus* sp. 1 Locality: APASM. Altitudinal record: 2200 m a.s.l. Low altitudinal specificity (IndVal: 0.17; p = 0.84).

#### *Parakiefferiella* Thienemann, 1936

44 valid species; worldwide; running and standing waters.

##### P. strixinorum

Taxon classificationAnimaliaDipteraChironomidae

Wiedenbrug & Andersen, 2002

###### Distribution.

BRAZIL – Taquara, Rio Grande do Sul State (29°46'S, 50°53'W); São Francisco de Paula, Rio Grande do Sul State (29°26'S, 50°35'W); Bom Jesus, Rio Grande do Sul State (28°40'S, 50°26'W).

###### Elevation.

600–1000 m a.s.l.

###### Habitats.

Stream.

###### Known stages.

P, M.

###### References.

Wiedenbrug and Andersen (2002).

###### Remarks.

*P. strixinorum* was found on marginal rocks of small streams, at 1045 m a.s.l., extending slightly the altitudinal range of the species. Environmental characterization: Water temperature 17 °C; dissolved oxygen 8.6 mg.l^-1^; pH 5; fast flowing; reduced vegetal canopy (less than 20% covered). The species was found in PESM (São Paulo State) extending the geographical records to northernmost. Low altitudinal specificity (IndVal: 0.17; p = 0.85).

**Unknown species.** *Parakiefferiella* sp. 1. Locality: PESM. Altitudinal record: 70 m a.s.l. Low altitudinal specificity (IndVal: 0.25; p = 0.33).

#### *Parametriocnemus* Goetgebuer, 1932

34 valid species; worldwide; springs, streams and rivers.

**Unknown species.** *Parametriocnemus* sp. 1. Locality: APASM. Altitudinal record: 2575 m a.s.l. High altitudinal specificity (IndVal: 0.33; p = 0.06); *Parametriocnemus* sp. 2. Locality: PESM and APASM. Altitudinal range: 25–1570 m a.s.l. Low altitudinal specificity (IndVal: 0.12; p = 0.70); *Parametriocnemus* sp. 3. Locality: PESM and APASM. Altitudinal range: 25–1445 m a.s.l. Low altitudinal specificity (IndVal: 0.11; p = 0.76).

#### *Pseudosmittia* Edwards, 1932

98 valid species; worldwide; aquatic, terrestrial and semi-terrestrial habitats.

##### P. catarinense

Taxon classificationAnimaliaDipteraChironomidae

Andersen, Saether & Mendes, 2010

###### Distribution.

BRAZIL – Parque Nacional de São Joaquim, Urubici, Santa Catarina State (28°07'32"S, 49°29'38"W).

###### Elevation.

1822 m a.s.l.

###### Habitats.

Male collected in malaise trap in cloud forest, close to small stream.

###### Known stages.

M.

###### References.

Andersen et al. (2010).

###### Remarks.

*P. catarinense* was found on rocky seepages, at 2200 m a.s.l., extending slightly it altitudinal occurrence. Environmental characterization: water temperature 27.6 °C; dissolved oxygen 7.0 mg.l^-1^; pH 6; slow flowing; vegetal coverage completely absent (shrub-herbaceous vegetation). The species was found in APASM (Minas Gerais State), extending the geographical records to northernmost. Low altitudinal specificity (IndVal: 0.17; p = 0.84).

#### *Rheocricotopus* Brundin, 1956

69 valid species; worldwide except Antarctica and Oceania; mostly rheophilic.

**Unknown species.** *Rheoricotopus* sp. 1 Locality: APASM. Altitudinal record: 2200 m. a.s.l. Low altitudinal specificity (IndVal: 0.17; p = 0.85); *Rheoricotopus* sp. 2. Locality: PARNASO. Altitudinal range: 1580–1670 m a.s.l. Median altitudinal specificity (IndVal: 0.29; p = 0.11).

#### *Urubicimbera* Andersen, Mendes & Pinho, 2015

One valid species; Brazil, Atlantic Forest; unknown habitats.

##### U. montana

Taxon classificationAnimaliaDipteraChironomidae

Andersen, Mendes & Pinho, 2015

###### Distribution.

BRAZIL – Parque Nacional de São Joaquim, Urubici, Santa Catarina State (28°07'37"S, 49°28'47"W).

###### Elevation.

1670 m a.s.l.

###### Habitats.

Male collected in malaise trap in cloud forest.

###### Known stages.

M.

###### References.

Andersen et al. (2015a).

###### Remarks.

*U. montana* was found on rocky seepages, from 2200–2700 m a.s.l., expanding its altitudinal range. Environmental characterization: Water temperature varied from 21.3–27.6 °C; dissolved oxygen 6.4–7.0 mg.l^-1^; pH 6; slow flowing; vegetal coverage completely absent (shrub-herbaceous vegetation). The species was found in APASM (Minas Gerais State), extending the geographical records to northernmost. Median altitudinal specificity (IndVal: 0.26; p = 0.13).

**Unknown species.** *Urubicimbera* sp. 1. Locality: APASM. Altitudinal range: 2575–2700 m a.s.l. Significant altitudinal specificity (IndVal: 0.91; **p = 0.002).**

### SUBFAMILY PODONOMINAE

#### TRIBE PODONOMINI

##### *Podonomus* Philippi, 1865

40 valid species; Neotropical and Australasian; running water and tarn inhabitants.

###### P. mina

Taxon classificationAnimaliaDipteraChironomidae

Shimabukuro, Pepinelli & Trivinho-Strixino, 2017

####### Remarks.

*P. mina* was recorded for the first time on marginal bedrock of a mountain stream, at 1270 m a.s.l. (20°25'12"S, 41°50'45.6"W), in Serra do Caparaó (Espírito Santo State) (Trivinho-Strixino et al. 2012), but only larvae have been evidenced and the description was only possible after the molecular association with the adults (Shimabukuro et al. 2017b). *P. mina* was recorded in isolated seepages and rocky substrate in the stream edges from 2575–2700 m a.s.l. in APASM (Minas Gerais State) (Shimabukuro et al. 2017b). Water temperature varied from 15–22 °C; dissolved oxygen 6.4–10.2 mg.l^-1^; pH 5.0–5.5; fast water flow; vegetal coverage completely absent Significant altitudinal specificity have been found (IndVal: 0.41; **p = 0.04).**

###### P. pepinellii

Taxon classificationAnimaliaDipteraChironomidae

Roque & Trivinho-Strixino, 2004

####### Distribution.

BRAZIL – Mantiqueira and Espinhaço mountain ranges: Monte Verde, Minas Gerais State (22°53'9.6"S, 46°1'55.2"W); Campos do Jordão, São Paulo State (22°46'1.2"S, 45°31'15.6"W); Teresópolis, Rio de Janeiro State (22°27'3.6"S, 43°0'50.4" W); Alto Caparaó, Minas Gerais State (20°25'12"S, 41°50'45.6"W).

####### Elevation.

1275–1815 m a.s.l.

####### Habitats.

Pupae found in a first-order stream; larvae found in madicolous habitats.

####### Known stages.

L, P, M, F.

####### References.

Roque and Trivinho-Strixino (2004); Trivinho-Strixino et al. (2012).

####### Remarks.

In this study larvae was found living on marginal rocks of a low order stream and in isolated rocky seepages, extending the altitudinal records up to 2700 m a.s.l. Environmental characterization: Water temperature varied from 10–22 °C; dissolved oxygen 6.4–9.0 mg.l^-1^; pH 5.0–6.0; very slow water flow; vegetal coverage completely absent (shrub-herbaceous vegetation). *P. pepinellii* was found in PARNASO (Rio de Janeiro State) and APASM (Minas Gerais State). Low altitudinal specificity (IndVal: 0.25; p = 0.32).

### SUBFAMILY TANYPODINAE

#### TRIBE PENTANEURINI

##### *Hudsonimyia* Roback, 1979

Four valid species; Nearctic and Neotropical; madicolous.

###### H. caissara

Taxon classificationAnimaliaDipteraChironomidae

Silva, Wiedenbrug, Trivinho-Strixino, Oliveira & Pepinelli, 2012

####### Distribution.

BRAZIL, Ubatuba, São Paulo State, (23°30.468'S, 45°11.923'W)

####### Elevation.

0 m a.s.l.

####### Habitats.

Few larvae found on leaf litter in shallow-water streams flowing over granite outcrops.

####### Known stages.

L, P, M.

####### References.

Silva et al. (2012).

####### Remarks.

*H. caissara* was found on marginal rocks of a small stream, at 200 m a.s.l. This slightly extended the altitudinal records of the species. Environmental characterization: Water temperature 21 °C; dissolved oxygen 7.9 mg.l^-1^; pH 5.5; fast flowing; sparse vegetal canopy (less than 30% covered). The species was found in PESM (São Paulo State). Median altitudinal specificity (IndVal: 0.33; p = 0.13).

**Unknown species.** *Hudsonimyia* sp.1. Locality: PESM. Altitudinal record: 1080 m a.s.l. Low altitudinal specificity (IndVal: 0.17; p = 0.85).

### Notes on altitudinal distribution

**Figure 3.**
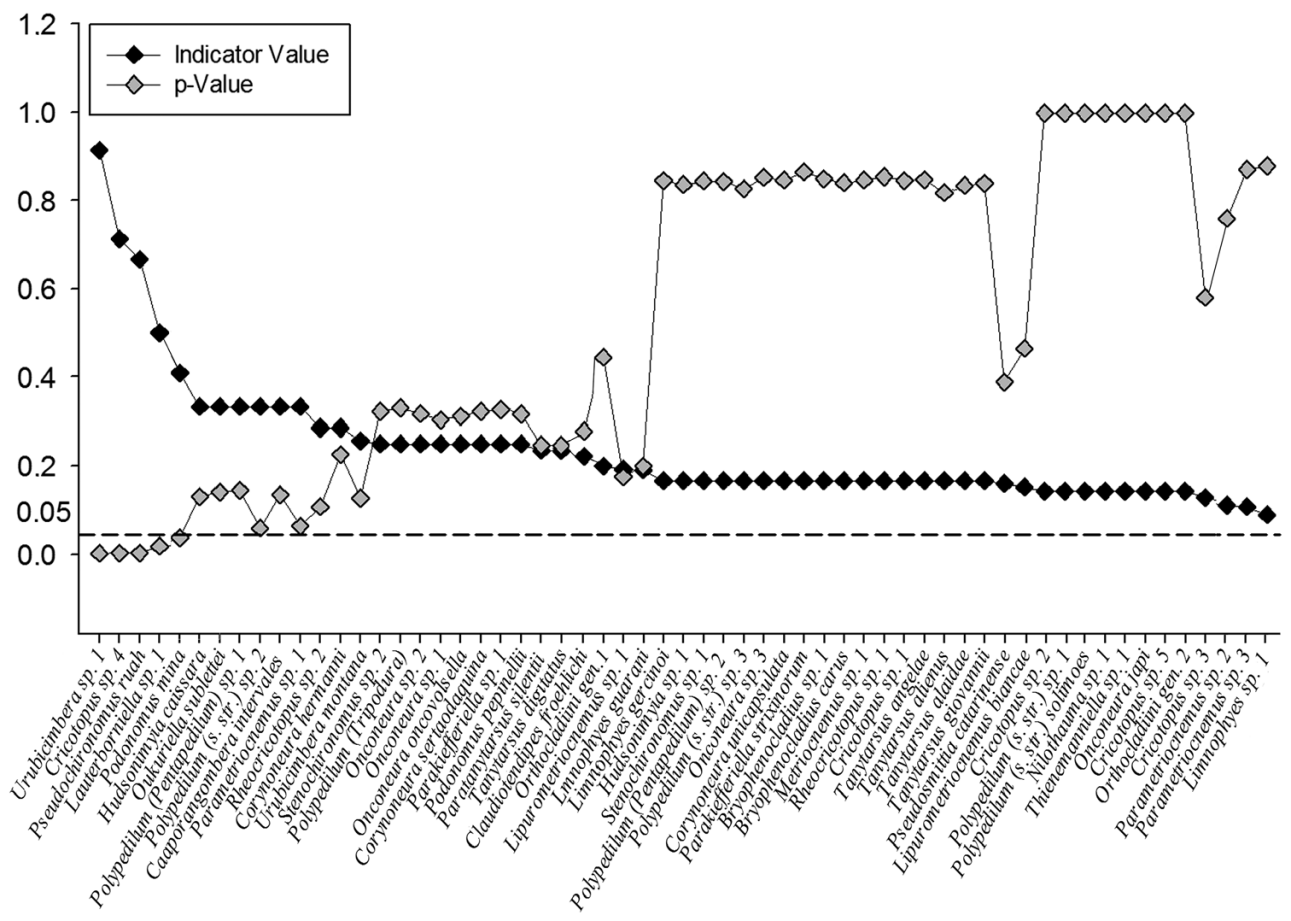
Indicator species of altitudinal range. Indicator values in black and significance value (p) at 0.05 level in gray obtained for each species and morphospecies found in the present study.

A summarized list of the species, morphospecies and the genera of immature found, along with respective ecological and geographical information, is presented in supplementary material (Table 1). In this study, the chironomid community was predominantly composed of species belonging to the subfamily Orthocladiinae (35 spp.), followed by Chironominae (21 spp), Podonominae and Tanypodinae (2 spp each). Among the 60 species recorded, a higher percentage has been found at APASM (45%), of which 85% were exclusive from this locality. Further, 31% of the possible new species occurred above 2100 m a.s.l. Only five from the 60 species recorded were significant indicators of specific altitudes, they are: *Urubicimbera* sp. 1, *Cricotopus* sp. 4, *Pseudochironomus ruah*, *Lauterborniella* sp. 1 and *Podonomus mina* (Figure 3). *Urubicimbera* sp. 1, and *P. mina*, represented the highest sites in this study (> 2600 m a.s.l.); *P. ruah* was a significant indicator of the 2500 m–altitudinal–band; *Lauterborniella* sp. 1 and *Cricotopus* sp. 4 were significant indicators of 1100 and 200 m–altitudinal–band, respectively (Figure 3). Furthermore, these five species were all unknown to science previous to this investigation in madicolous habitats of the Atlantic Forest.

**Figure 4.**
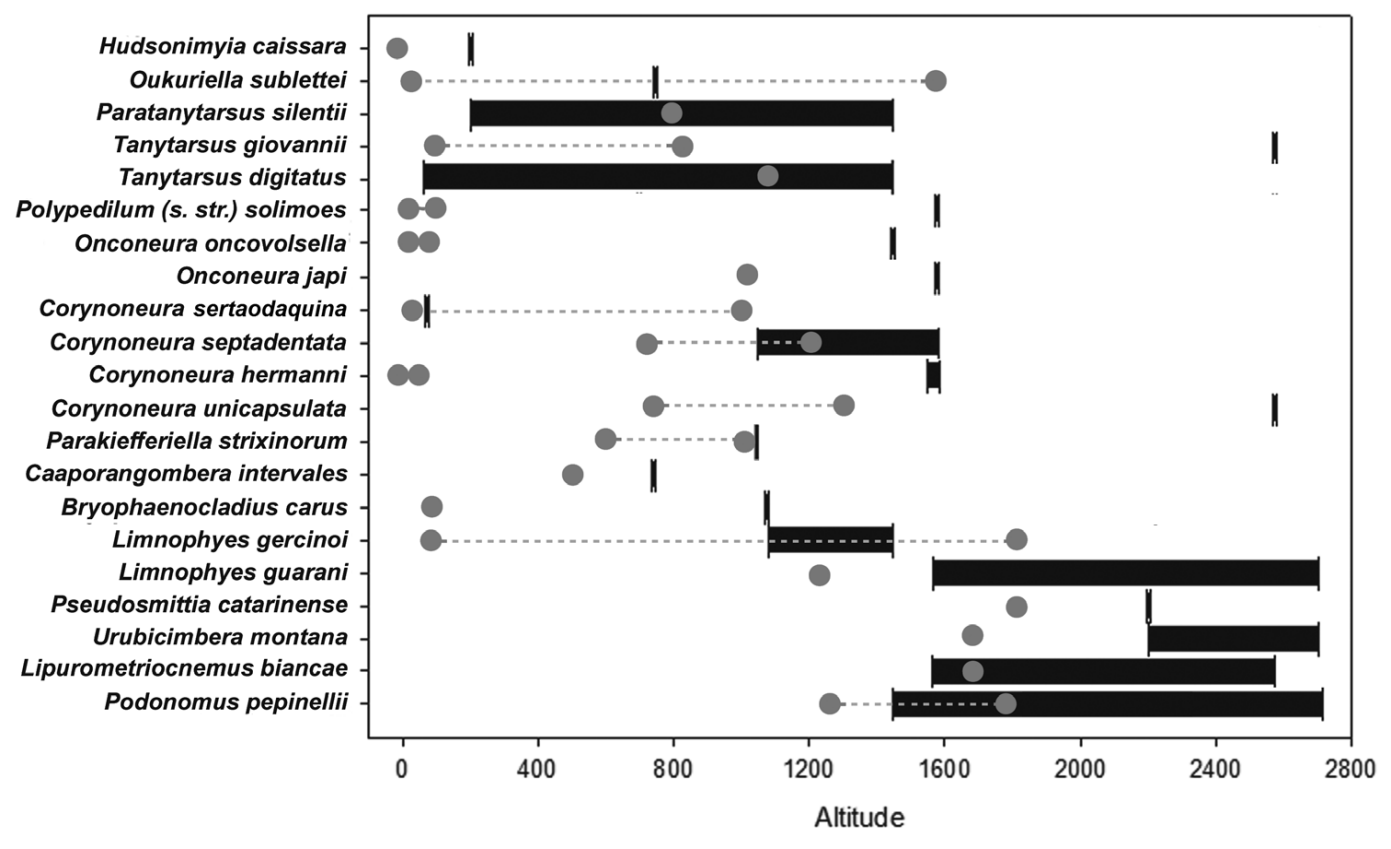
Altitudinal record of each species found in madicolous habitats in the present study. Previous altitudinal records (from literature) in gray and altitudinal records from this research in black.

Regarding the 22 recognized species, 17 of them had spread the altitudinal distribution (Figure 4). Even those species that have previously been found in mountain regions, such as *Podonomus pepinellii*, *Lipurometriocnemus biancae*, *Urubicimbera montana*, *Pseudosmittia catarinense* and *Limnophyes guarani*, were recorded at higher altitudes in this study, and, except for *P. catarinense*, the altitudinal distribution increased more than 1000 m for each of these species (Figure 4).

For those species that have so far been verified at low altitudes, such as *Tanytarsus giovannii*, *Polypedilum solimoes*, *Onconeura oncovolsella*, *Corynoneura hermanni* and *Bryophaenocladius carus*, the amplitude of the altitudinal distribution was even more remarkable, adding more than 1500 m to the altitudinal range in some cases. The only exception was *Hudsonimyia caissara*, that have been firstly reported at the sea level, and here it was found at 200 m a.s.l., slightly increasing the altitudinal range of the species (Figure 4). *Paratanytarsus silentii*, *Tanytarsus digitatus*, *Onconeura japi*, *Corynoneura septadentata*, *Corynoneura unicapsulata* and *Caaporangombera intervales* also had the altitudinal distribution extended, while the remaining five species were recorded within their typical altitudinal ranges (Figure 4).

## Discussion

Compositional changing in chironomid assemblages along altitudinal gradients have been verified by many researchers worldwide (McKie et al. 2005, Tejerina and Malizia 2012, Henriques-Oliveira and Nessimian 2010, Scheibler et al. 2014, Robinson et al. 2016, Matthews-Bird et al. 2016). In mountain ecosystems the occurrence of chironomid species can be influenced by environmental changes related to altitude variation, such as temperature and oxygen availability (Oliver 1971, Pinder 1986, Eggermont and Heiri 2012), dispersal capacity (Ashe et al. 1987), historical events (McKie et al. 2005, Allegrucci et al. 2006, Krosch et al. 2011) or other regional particularities (Körner 2007). Mountains are therefore important objects to biogeography studies, revealing rich communities and many endemic species (Lods-Crozet et al. 2001, Garcia and Suaréz 2007; Brundin 1966). In our study, the locality with higher altitudes (APASM) yielded a higher number of species, most of them are unknown to science and were exclusively found in this place, especially above 2,100 m a.s.l.. These evidences are essential in view of the conservation perspective, once the majority of the species found are possibly endemic.

A clear gap on the taxonomic knowledge of mountain fauna can be observed. This gap is likely due to the low accessibility of these areas, thereby hampering sampling strategies. Studies in mountain regions are urgently needed, especially when dealing with one of the most threatened biomes in the world (Ribeiro et al. 2009) whose geomorphological characteristics are so heterogeneous. Mountains have been suffering from several types of environmental impacts, but the most alarming today is climate change (Burke 2003, Catalan et al., 2017). Current forecasts suggest that rainfall will be less constant and temperature will raise 2.0 to 6.0 °C by 2100 (Garcia et al. 2014), strongly affecting the flora and fauna. The climate changes will especially affect those living in small water bodies with high exposure to environmental pressure. The narrow range of tolerance to environmental conditions verified for mountain species, and the fact that many of them are rare and endemic, make the conservation efforts in these areas indispensable.

The indicator's analysis evidenced that all species significantly associated with their respective altitudinal band were previously unknown to science. All of them, except for *Cricotopus* sp. 4, recorded from 70–200 m a.s.l. were found exclusively at mountaintops. *Lauterborniella* sp. 1 was recorded at the highest sites in PESM and the remaining taxa were recorded at the summit of APASM mountains. Regarding the geophysical characteristics of mountaintops (shape, size, insulation value), also known as Inselbergs (Porembski 2007); some species, especially those with limited dispersal capacity, are more likely to deal with speciation process and local extinctions (MacArthur and Wilson, 1967). The narrow altitudinal range expressed by these unknown species, make us believe that they should present a high endemicity degree.

Our new records extend the altitudinal range of 17 known species. Most species seem to tolerate a wide altitudinal range, such as *Tanytarsus giovannii* and *Limnophyes gercinoi*, while others presented a narrow range, such as *Hudsonimyia caissara*. The altitudinal range is related to the extent of the geographical distribution of each species; species that are widely distributed are expected to occur in a wider range of altitudes compared to those that have limited distribution (Stevens 1992). Brundin (1966), analyzing the distributional patterns of Podonominae in South America concluded that species found in Patagonian region could be recorded at the highest sites of tropical Andean mountains. Similarly, in this study, species that have previously been recorded further south such as *Lipurometriocnemus biancae*, *Urubicimbera montana*, *Pseudosmittia catarinense*, and *Limnophyes guarani*, were found at higher altitudes, and may be related to temperature requirements.

Madicolous habitats have never been formally studied in Brazilian mountainous regions, in contrast to other Atlantic Forest water bodies in which the Chironomidae fauna have already been extensively investigated (Henriques-Oliveira et al. 2003, Roque et al. 2007, Silveira et al. 2015). Taxonomists, and especially ecologists, have paid little attention to semi-aquatic and terrestrial Chironomidae, and therefore, madicolous species were completely overlooked. In our current study, a remarkable diversity of Chironomidae living in madicolous habitats was revealed, and most of the species (about 64%) were probably new before this project. However, from the 38-unknown species collected, five have recently been described: *T. alaidae, T. alienus, T. angelae* (Trivinho-Strixino and Shimabukuro 2017), *P. mina* (Shimabukuro et al. 2017b), and *P. ruah* (Shimabukuro et al. 2017a), increasing the number of madicolous chironomid species.

Despite the low knowledge on semi-aquatic forms, evidences from chironomids fossils preserved in amber reveals that terrestrial life-styles have been common since the late Eocene (about 40 million years ago) (Zelentsov et al. 2012), raising the importance of madicolous and other semi-aquatic habitats on the evolutionary history of many Chironomidae taxa. Within them, Orthocladiinae harbors the majority of semi-aquatic species (Andersen et al. 2010), what might explain their notable richness in madicolous habitats. Sinclair and Marshall (1987) also noted the remarkable dominance of Orthocladiinae among madicolous chironomids in Southern Ontario, Canada. In their study, ten of 14 genera recorded were Orthocladiinae, including *Parakiefferiella*, *Metriocnemus*, *Parametriocnemus*, *Thienemanniella*, and *Limnophyes*, also verified in this study. One more evidence that these genera can adapt well to this habitat.

Only two of the species verified here were previously known to occur in madicolous habitats (*Podonomus pepinellii* and *Limnophyes guarani*). Although *Podonomus* larvae can be found in streams and other fast flowing running waters, they are also common on the edge of streams (Brundin 1966). *Podonomus pepinellii* and all *Podonomus* morphotypes in the Atlantic Forest highlands are associated with madicolous habitats (Trivinho-Strixino et al. 2012), and as such they occur in rocky outflows and stream shorelines. The remaining species identified in this study were previously considered stream-dweller, although some have been found in habitats close to madicolous ones, such as those from the Corynoneurini tribe and *Hudsonimyia caissara*.

The larvae of *Hudsonimyia caissara* were originally found in low abundance (two specimens) in leaf litter of a mountain stream (Silva et al. 2012), possibly an inhabitant of the stones in the stream's edge. Further, it is very plausible that some stream-dweller species can tolerate both conditions (Vaillant 1956, Sinclair and Marshall 1987). Thus, a richer fauna is expected to occur in marginal stream rocks compared to isolate seepages.

It is not surprising that members of *Hudsonimyia*, *Bryophaenocladius*, *Metriocnemus*, *Limnophyes* and *Pseudosmittia* have been found in madicolous habitats during this study. These genera are known to have larval instars associated with semi-aquatic and terrestrial conditions. Roback (1979) was the first to verify *Hudsonimyia* larvae living on a thin layer of current water with periphyton and moss. *Metriocnemus* species are adapted to an extremely broad range of habitats within Chironomidae (Cranston and Judd 1987), including madicolous, as exemplified by the species *M. hygropetricus* (Kieffer 1911), whose name was given after their type locality habitat – natural rock seepages and artificial madicoulous habitats. Most *Limnophyes* larvae are semi-aquatic (Saether 1990), and recently a new species of this genus, *Limnophyes guarani*, has been recorded on madicolous habitats in the south of Brazil (Pinho and Andersen 2015). *Pseudosmittia* and *Bryophaenocladius* larvae are largely terrestrial or semi-terrestrial; however, this was the first time that Neotropical species of these genera have been recorded in a madicolous habitat.

Although many genera were expected to occur, some were particularly intriguing, such as *Stenochironomus* and *Oukuriella*. Both are known to be highly habitat-specialized in larval phase. The first is a vegetal miner (Epler et al. 2013) while the second is typically associated with sponge or wood detritus, although the habitat of basal groups in the phylogeny of *Oukuriella* could not be defined yet (Fusari et al. 2014). *Oukuriella sublettei* recorded in this study was reported in association with submerged wood found in first order streams with bedrock (Bellodi et al. 2016). Their presence in the marginal rocks of the stream might have been accidental, considering that only one specimen was found. The same is expected for both *Stenochironomus* species. Their reduced size suggests that adults emerged from leaf detritus or small fragments of wood, possibly inside rock fissures. However, the emergence of these taxa in such conditions was interesting, since in this case they completed their development in a thin layer of water, a complement to previous observations of immature submerged in the streams (Fusari et al. 2014, Bellodi et al. 2016; Dantas et al. 2016).

Far from being semi-aquatic, most of the *Rheotanytarsus* species require flowing water conditions to survive and emerge (Coffman and Ferrington 1996). However, the capacity to live in madicolous habitats may not be disregarded as some species, such as *R. gloveri*, demonstrated tolerance to drying rock faces of streams and survived in thin layers of current water (Cranston 1997). The strict definition given by Vaillant, in 1956, considers hygropetric habitats all flowing water with less than 2 mm thick. However, this delimitation is hard to establish when dealing with microhabitats constantly susceptible to water flow oscillations due to climatic conditions. In some occasions, our sampling sites, especially those at the margins of the streams, had the water flux modified as a consequence of the contraction or expansion of the main channel. Probably, *Rheotanytarsus* species were favored when stronger currents rose, although the intense dark coloration of the cephalic capsule of the larvae may indicate that they are truly madicolous inhabitants (Brundin 1966, Sinclair 1988, Trivinho-Strixino et al. 2012).

The procedure of rearing immature specimens to obtain the adults is most of the time unsuccessful due to their environmental requirements (Ekrem et al. 2007). Therefore, descriptions are frequently based only on adults, whose sampling methods often preclude the knowledge of immature habitat and other aspects of their ecology. Some species recorded have only been known by the adults, previously sampled with malaise or light traps. This was the case of *Lipurometriocnemus biancae*, *Urubicimbera montana*, *Pseudosmittia catarinense*, *Caaporangombera intervales*, and *Paratanytarsus silentii*.

Using our modified emergence traps allowed us to assure that the immature organisms and the adults from the species sampled in this study were madicolous inhabitants. The association and description of the immature is a fundamental task when studying chironomids, best accomplished with the help of molecular tools, such as DNA barcode. The larva and the pupa of *P. silentii* have been successfully associated with adult males after this investigation (Trivinho-Strixino and Shimabukuro 2017). Furthermore, even for those species whose immature forms are known, the first record of them in madicolous habitats represents a remarkable note on their success in colonizing a wide range of habitats.

## Supplementary Material

XML Treatment for C. froehlichi

XML Treatment for O. sublettei

XML Treatment for P. solimoes

XML Treatment for P. ruah

XML Treatment for P. silentii

XML Treatment for T. alaidae

XML Treatment for T. alienus

XML Treatment for T. angelae

XML Treatment for T. digitatus

XML Treatment for T. giovannii

XML Treatment for C. unicapsulata

XML Treatment for C. hermanni

XML Treatment for C. septadentata

XML Treatment for C. sertaodaquina

XML Treatment for O. japi

XML Treatment for O. oncovolsella

XML Treatment for B. carus

XML Treatment for C. intervales

XML Treatment for L. guarani

XML Treatment for L. gercinoi

XML Treatment for L. biancae

XML Treatment for P. strixinorum

XML Treatment for P. catarinense

XML Treatment for U. montana

XML Treatment for P. mina

XML Treatment for P. pepinellii

XML Treatment for H. caissara

## Supplementary materials

Supplementary material 1 Table S1. Taxa recorded in madicolous habitats from Atlantic Forest in Southeastern Brazil Data type: species data Explanation note: DS: development stage; A: adults; I: immature; SE: stream edges; RS: rocky seepages; LAB: low altitudinal band, sampling sites in PESM; IAB: intermediate altitudinal band, sampling sites in PARNASO; HAB: high altitudinal band, sampling sites in APASM. This is an open access article distributed under the terms of the Creative Commons Attribution License (CC BY 4.0), which permits unrestricted use, distribution, and reproduction in any medium, provided the original author and source are credited.Erika Mayumi Shimabukuro, Susana Trivinho-Strixino
