# Supplementary material for: Madicolous Chironomidae from the Brazilian Atlantic Forest: a checklist with notes on altitudinal distributions (Diptera, Insecta)
Source: Zookeys. 2018 Apr 19;(751):41–73. doi: 10.3897/zookeys.751.20611 (PMC5919911; doi:10.3897/zookeys.751.20611)
Supplement: Supplementary material 1 — Table S1. Taxa recorded in madicolous habitats from Atlantic Forest in Southeastern Brazil [file zookeys-751-041-s001.docx]

Supplementary material

**TABLE 1.** Taxa recorded in madicolous habitats from Atlantic Forest in Southeastern Brazil. DS: development stage; A: adults; I: immature; SE: stream edges; RS: rocky seepages; LAB: low altitudinal band, sampling sites in PESM; IAB: intermediate altitudinal band, sampling sites in PARNASO; HAB: high altitudinal band, sampling sites in APASM.

|  | Development stage | | Habitat | | Altitudinal Band | | |
| --- | --- | --- | --- | --- | --- | --- | --- |
|  | I | A | SE | RS | LAB | IAB | HAB |
| **CHIRONOMINAE** |  |  |  |  |  |  |  |
| **Chironomini** |  |  |  |  |  |  |  |
| *Beardius* sp. | x |  | x |  | x |  | x |
| *Chironomus* sp*.* | x |  | x |  | x |  |  |
| *Claudiotendipes froehlichi* Andersen, Mendes & Pinho, 2017 |  | x | x |  | x | x |  |
| *Lauterborniella* sp. 1* |  | x | x |  | x |  |  |
| *Nilothauma* sp. 1* |  | x | x |  |  |  | x |
| *Oukuriella sublettei* Messias & Oliveira, 1999 |  | x | x |  | x |  |  |
| *Phaenospectra* sp. | x |  | x |  | x |  | x |
| *Polypedilum* *(Pentapedilum)* sp 1* |  | x | x |  | x |  |  |
| *Polypedilum* *(Pentapedilum)* sp 2* |  | x | x |  |  | x |  |
| *Polypedilum (s. str.) solimoes* Bidawid-Kafka, 1996 |  | x | x |  |  |  | x |
| *Polypedilum* *(s. str.)* sp 1* |  | x | x |  |  |  | x |
| *Polypedilum* *(s. str.)* sp 2* |  | x | x |  | x |  |  |
| *Polypedilum* *(s. str.)* sp 3* |  | x | x |  |  | x |  |
| *Polypedilum (Tripodura)* sp. 1*** |  | x | x |  | x |  |  |
| *Stenochironomus* sp. 1* |  | x | x |  | x |  |  |
| *Stenochironomus* sp. 2* |  | x | x |  | x |  |  |
| Chironomini Gênero 1 | x |  | x |  | x | x | x |
| **Pseudochironomini** |  |  |  |  |  |  |  |
| *Pseudochironomus ruah* Shimabukuro & Trivinho-Strixino, 2017 | x | x | x |  |  |  | x |
|  | Development stage | | Habitat | | Altitudinal Band | | |
|  | I | A | SE | RS | LAB | IAB | HAB |
| *Riethia* sp. | x |  | x | x |  | x | x |
| **Tanytarsini** |  |  |  |  |  |  |  |
| *Constempellina* sp. | x |  | x |  | x |  | x |
| *Paratanytarsus silentii* Trivinho-Strixino, 2010 | x | x | x |  | x | x |  |
| *Rheotanytarsus* sp. | x |  | x | x | x | x | x |
| *Stempelinella* sp. | x |  | x |  | x |  |  |
| *Tanytarsus alaidae* Trivinho-Strixino & Shimabukuro, 2017 |  | x | x |  |  |  | x |
| *Tanytarsus alienus* Trivinho-Strixino & Shimabukuro, 2017 |  | x | x |  |  |  | x |
| *Tanytarsus angelae* Trivinho-Strixino & Shimabukuro, 2017 |  | x | x | x |  | x | x |
| *Tanytarsus digitatus* Sanseverino & Fittkau, 2006 |  | x | x |  | x | x |  |
| *Tanytarsus giovannii* Sanseverino & Trivinho-Strixino, 2010 |  | x | x |  |  |  | x |
| **ORTHOCLADIINAE** |  |  |  |  |  |  |  |
| *Antilocladius* sp. | x |  | x |  | x | x | x |
| *Bryophenocladius carus* Roback 1962 |  | x | x |  | x |  |  |
| *Bryophenocladius* sp. 1* |  | x | x |  | x |  |  |
| *Caaporangombera intervales* Andersen, Pinho & Mendes, 2015 |  | x | x |  | x |  |  |
| *Corynoneura hermanni* Wiedenbrug & Trivinho-Strixino, 2011 |  | x | x | x |  | x | x |
| *Corynoneura septadentata* Wiedenbrug & Trivinho-Strixino, 2011 |  | x | x | x | x | x |  |
| *Corynoneura sertaodaquina* Wiedenbrug & Trivinho-Strixino, 2011 |  | x | x |  | x |  |  |
| *Corynoneura unicapsulata* Wiedenbrug & Trivinho-Strixino, 2011 |  | x | x |  |  |  | x |
| *Cricotopus* sp 1* |  | x | x |  |  |  | x |
| *Cricotopus* sp 2* |  | x | x |  |  | x | x |
| *Cricotopus* sp 3* |  | x | x |  | x |  |  |
| *Cricotopus* sp 4* |  | x | x |  | x |  | x |
| *Cricotopus* sp 5* |  | x | x |  |  |  | x |
| *Gymnometriocnemus* sp. | x |  | x |  |  | x | x |
|  | Development stage | | Habitat | | Altitudinal Band | | |
|  | I | A | SE | RS | LAB | IAB | HAB |
| *Limnophyes gercinoi* (Oliveira, Messixs & Santos, 1995) |  | x | x |  | x | x |  |
| *Limnophyes guxrxni* Pinho & Andersen, 2015 |  | x | x | x |  |  | x |
| *Limnophyes* sp. 1* |  | x | x |  |  | x |  |
| *Lipurometriocnemus bixncxe* Andersen, Pinho & Mendes, 2016 |  | x | x |  |  | x | x |
| *Lipurometriocnemus* sp. 1* |  | x |  | x |  |  | x |
| *Lopesclxdius* sp. | x |  | x |  | x |  | x |
| *Metriocnemus* sp. 1* |  | x |  | x |  |  | x |
| *Nxnoclxdius* sp. | x |  | x |  |  |  | x |
| *Onconeura japi* Wiedenbrug, Mendes, Pepinelli & Trivinho-Strixino, 2009 |  | x | x |  |  |  | x |
| *Onconeura oncovolsella* Wiedenbrug, Mendes, Pepinelli & Trivinho-Strixino, 2009 |  | x | x |  |  | x |  |
| *Onconeura* sp. 1* |  | x | x |  |  | x |  |
| *Onconeura* sp. 2* |  | x | x |  |  | x |  |
| *Onconeura* sp. 3* |  | x | x |  | x |  |  |
| *Parakiefferiella* sp. 1* |  | x | x |  | x |  |  |
| *Parakiefferiella strixinorum* Wiedenbrug & Andersen, 2002 |  | x | x |  | x |  |  |
| *Parametriocnemus* sp. 1* |  | x | x |  |  |  | x |
| *Parametriocnemus* sp. 2* |  | x | x |  | x |  | x |
| *Parametriocnemus* sp. 3* |  | x | x |  | x | x |  |
| *Paraphaenocladius* sp. | x |  | x | x | x | x | x |
| *Psectrocladius* sp. | x |  | x | x |  | x | x |
| *Pseudosmittia catarinense* Andersen, Sæther & Mendes, 2010 |  | x |  | x |  |  | x |
| *Rheocricotopus* sp. 1* |  | x |  | x |  |  | x |
| *Rheocricotopus* sp. 2* |  | x | x |  |  | x |  |
| *Thienemannia* sp. | x |  | x | x |  | x | x |
| *Thienemanniella* sp. 1* |  | x | x |  |  |  | x |
| *Ubatubaneura* | x |  | x |  | x |  |  |
|  | Development stage | | Habitat | | Altitudinal Band | | |
|  | I | A | SE | RS | LAB | IAB | HAB |
| *Urubicimbera montana* Andersen, Mendes & Pinho, 2015 |  | x |  | x |  |  | x |
| *Urubicimbera* sp. 1* |  | x | x | x |  |  | x |
| Orthocladiini Gênero 1 |  | x |  | x |  |  | x |
| Orthocladiini Gênero 2 |  | x | x |  |  | x |  |
| **PODONOMINAE** |  |  |  |  |  |  |  |
| **Podonomini** |  |  |  |  |  |  |  |
| *Podonomus* *mina* Shimabukuro, Pepinelli & Trivinho-Striaino, 2017 | x | x | x | x |  |  | x |
| *Podonomus pepinellii* Roque & Trivinho-Striaino, 2004 |  | x | x | x |  | x | x |
| **TANYPODINAE** |  |  |  |  |  |  |  |
| **Macropelopini** |  |  |  |  |  |  |  |
| *Alotanypus* sp. | x |  | x |  |  |  | x |
| **Pentaneurini** |  |  |  |  |  |  |  |
| *Ablabesmyia* sp. | x |  | x |  |  |  | x |
| *Hudsonimyia caissara* Silva, Wiedenbrug, Trivinho-Striaino, Oliveira & Pepinelli, 2012 |  | x | x |  | x |  |  |
| *Hudsonimyia* sp. 1* |  | x | x |  | x |  |  |
| *Larsia* sp. | x |  | x | x | x |  | x |
| *Nilotanypus* sp. | x |  | x |  |  |  | x |
| *Parapentaneura* sp. | x |  | x | x | x | x |  |
| *Pentaneura* sp. | x |  | x | x | x | x | x |
| *Thienemanimyia* sp. | x |  | x |  | x |  | x |
| **Procladini** |  |  |  |  |  |  |  |
| *Djalmabatista* sp. | x |  | x |  |  |  | x |

*species unknown to science
